# Supplementary material for: Unveiling the Neem (Azadirachta indica) Effects on Biofilm Formation of Food-Borne Bacteria and the Potential Mechanism Using a Molecular Docking Approach
Source: Plants (Basel). 2024 Sep 23;13(18):2669. doi: 10.3390/plants13182669 (PMC11434743; doi:10.3390/plants13182669)
Supplement: Supplementary file 1 [file plants-13-02669-s001.zip › plants-3176128-supplementary.pdf]

**Table S1.** Phytochemical analysis of methanolic leaf extract of *Azadirachta indica* using gas chromatography-mass spectrometry.

| S. No. | RT | Name of the compound | Molecular Formula | Molecular weight | Peak area (%) |
|---|---|---|---|---|---|
| 1. | 34.4 | D-Glucose, 4-O-à-D-glucopyranosyl | $C_{12}H_{22}O_{11}$ | 342 | 13.03 |
| 2. | 36.93 | Phytol | $C_{20}H_{40}O$ | 296 | 8.31 |
| 3. | 20.63 | 5-(1-Ethoxy-ethoxy)-4-methyl-hex-2-enal | $C_{11}H_{20}O_3$ | 200 | 6.73 |
| 4. | 19.7 | Heptasiloxane-tetradecamethyl | $C_{14}H_{44}O_6Si_7$ | 504 | 4.86 |
| 5. | 24.13 | Cycloheptasiloxane, tetradecamethyl | $C_{14}H_{42}O_7Si_7$ | 518 | 4.4 |
| 6. | 37.35 | Pseudosolasodine diacetate | $C_{31}H_{49}NO_4$ | 499 | 3.32 |
| 7. | 36.71 | 5,8,11,14-Eicosatetraenoic acid, methyl ester | $C_{21}H_{34}O_2$ | 318 | 2.95 |
| 8. | 42.18 | Phenol, 2,2'-methylenebis[6-(1,1-dimethylethyl)-4-methy | $C_{23}H_{32}O_2$ | 340 | 2.85 |
| 9. | 14.18 | Dihydroxy-2,5-dimethyl-3(2H)-furan-3-one | $C_6H_8O_4$ | 144 | 2.58 |
| 10. | 34.53 | Glyceryl monolinoleate | $C_{27}H_{54}O_4Si_2$ | 498 | 2.42 |
| 11. | 34.53 | 1-Monolinoleoylglycerol trimethylsilyl ether | $C_{27}H_{54}O_4Si_2$ | 498 | 2.42 |
| 12. | 34.03 | Estra-1,3,5(10)-trien-17á-ol | $C_{18}H_{24}O$ | 256 | 2.17 |
| 13. | 34.05 | l-(+)-Ascorbic acid 2,6-dihexadecanoate | $C_{38}H_{68}O_8$ | 652 | 2.17 |
| 14. | 28.08 | Cyclooctasiloxane, hexadecamethyl | $C_{16}H_{48}O_8Si_8$ | 295 | 2.14 |
| 15. | 29.79 | Octahydro-2(1H)-quinolinone | $C_9H_{15}NO$ | 153 | 2.08 |
| 16. | 33.39 | Hexadecanoic acid, methyl ester | $C_{17}H_{34}O_2$ | 270 | 2.08 |
| 17. | 31.49 | Heptasiloxane, hexadecamethyl | $C_{16}H_{48}O_6Si_7$ | 532 | 1.64 |
| 18. | 36.38 | Oleic Acid | $C_{18}H_{34}O_2$ | 282 | 1.39 |
| 19. | 16.55 | Benzofuran, 2,3-dihydro | $C_8H_8O$ | 120 | 1.38 |
| 20. | 52.38 | 5à-Pregn-16-en-20-one, 3á,12à-dihydroxy-diacetate | $C_{25}H_{36}O_5$ | 416 | 1.38 |
| 21. | 39.87 | Glycine, N-[(3à,5á,7à,12à)-24-oxo-3,7,12tris[(trimethylsilyl)oxy] cholan-24-yl]-, methyl ester | $C_{36}H_{69}NO_6Si_3$ | 695 | 1.27 |
| 22. | 52.04 | Carotene, 1,1',2,2'-tetrahydro-1,1'-dimethoxy | $C_{42}H_{64}O_2$ | 600 | 0.9 |
| 23. | 43.50 | Dasycarpidan-1-methanol, acetate (ester) | $C_{20}H_{26}N_2O_2$ | 326 | 0.85 |
| 24. | 40.76 | 9,10-Secocholesta-5,7,10(19)-triene-1,3-diol, 25-[(trimethylsilyl)oxy]-, (3á,5Z,7E) | $C_{30}H_{52}O_3Si$ | 488 | 0.8 |
| 25. | 19.3 | 2-Methoxy-4-vinylphenol | $C_9H_{10}O_2$ | 150 | 0.69 |
| 26. | 18.13 | Ergosta-5,22-dien-3-ol, acetate | $C_{30}H_{48}O_2$ | 440 | 0.65 |

| | | | | | |
|---|---|---|---|---|---|
| **27.** | 43.04 | Ethyl 5,8,11,14,17-icosapentaenoate | $C_{22}H_{34}O_2$ | 330 | 0.65 |
| **28.** | 26.84 | à-D-Glucopyranoside, methyl 2-(acetylamino)-2-deoxy-3-O-(trimethylsilyl)-, cyclic methylboronate | $C_{13}H_{26}BNO_6Si$ | 331 | 0.59 |
| **29.** | 17.13 | Octadecanoic acid, 3-hydroxy-, methyl ester | $C_{19}H_{38}O_3$ | 314 | 0.58 |
| **30.** | 24.49 | Ricinoleic acid | $C_{18}H_{34}O_3$ | 298 | 0.58 |
| **31.** | 4.6 | 1,3,3-Trimethoxybutane | $C_7H_{16}O_3$ | 148 | 0.51 |
| **32.** | 23.57 | 12-Octadecenal | $C_{18}H_{34}O$ | 266 | 0.55 |
| **33.** | 42.77 | Pregn-4-ene-3,20-dione, 17,21-dihydroxy-, bis(O-methyloxime) | $C_{23}H_{36}N_2O_4$ | 404 | 0.39 |
| **34.** | 38.61 | 1-Heptatriacotanol | $C_{37}H_{76}O$ | 536 | 0.32 |
| **35.** | 43.24 | Ursodeoxycholic acid | $C_{24}H_{40}O_4$ | 392 | 0.32 |
| **36.** | 20.31 | 2,4-Dimethoxyphenol | $C_8H_{10}O_3$ | 145 | 0.3 |
| **37.** | 24.94 | Cholestan-3-ol, 2-methylene-, (3á,5à) | $C_{28}H_{48}O$ | 400 | 0.29 |
| **38.** | 4.78 | Geranyl isovalerate | $C_{15}H_{26}O_2$ | 238 | 0.27 |
| **39.** | 10.68 | Sarreroside | $C_{30}H_{42}O_{10}$ | 562 | 0.18 |
| **40.** | 41.58 | Retinol | $C_{20}H_{30}O$ | 286 | 0.18 |
| **41.** | 36.38 | Erucic acid | $C_{22}H_{42}O_2$ | 338 | 0.17 |
| **42.** | 5.03 | 5,7-Dodecadiyn-1,12-diol | $C_{12}H_{18}O_2$ | 194 | 0.16 |
| **43.** | 38.32 | Linoleic acid ethyl ester | $C_{20}H_{36}O_2$ | 308 | 0.13 |
| **44.** | 16.92 | Pyrimidine-2,4(1H,3H)-dione, 1,3-dimethyl-6-[2-(4-morpholyl)ethenyl]-5-nitro | $C_{12}H_{16}N_4O_5$ | 296 | 0.11 |
